# Phospholipids and Fatty Acids Affect the Colonization of Urological Catheters by *Proteus mirabilis*

**DOI:** 10.3390/ijms22168452

**Published:** 2021-08-06

**Authors:** Paulina Stolarek, Przemysław Bernat, Dominika Szczerbiec, Antoni Różalski

**Affiliations:** 1 Department of Biology of Bacteria, Faculty of Biology and Environmental Protection, University of Lodz, Banacha 12/16, 90-237 Lodz, Poland; dominika.szczerbiec@biol.uni.lodz.pl (D.S.); antoni.rozalski@biol.uni.lodz.pl (A.R.); 2 Department of Industrial Microbiology and Biotechnology, Faculty of Biology and Environmental Protection, University of Lodz, Banacha 12/16, 90-237 Lodz, Poland; przemyslaw.bernat@biol.uni.lodz.pl

**Keywords:** *Proteus mirabilis*, adhesion, catheter, inner membrane potential, cell-surface hydrophobicity, generalized polarization, fatty acids, phospholipids, phosphatidylethanolamines, phosphatidylglycerols

## Abstract

*Proteus mirabilis*-mediated CAUTIs are usually initiated by the adherence of bacteria to a urinary catheter surface. In this paper, three isolates of different origin and exhibiting different adhesion abilities were investigated in search of any changes in lipidome components which might contribute to *P. mirabilis* adhesion to catheters. Using GC-MS and LC-MS/MS techniques, 21 fatty acids and 27 phospholipids were identified in the examined cells. The comparison of the profiles of phospholipids and fatty acids obtained for catheter-attached cells and planktonic cells of the pathogens indicated C11:0 and PE 37:2 levels as values which could be related to *P. mirabilis* adhesion to a catheter, as well as *cis* C16:1, PE 32:0, PE 33:0, PE 38:2, PG 33:1, PG 34:0, PE 30:1, PE 32:1 and PG 30:2 levels as values which could be associated with cell hydrophobicity. Based on DiBAC_4_ (3) fluorescence intensity and an affinity to *p*-xylene, it was found that the inner membrane depolarization, as well as strong cell-surface hydrophobicity, were important for *P. mirabilis* adhesion to a silicone catheter. A generalized polarization of Laurdan showed lower values for *P. mirabilis* cells attached to the catheter surface than for planktonic cells, suggesting lower packing density of membrane components of the adherent cells compared with tightly packed, stiffened membranes of the planktonic cells. Taken together, these data indicate that high surface hydrophobicity, fluidization and depolarization of *P. mirabilis* cell membranes enable colonization of a silicone urinary catheter surface.

## 1. Introduction

*Proteus mirabilis* is a Gram-negative, dimorphic bacterium, belonging to the *Morganellaceae* family (formerly classified as *Enterobacteriaceae*) [1], known for numerous and varied virulence factors, such as invasiveness, swarming motility, urease, proteases and amino acid deaminases activities, synthesis of flagella and fimbriae, and production of hemolysins, capsular polysaccharide and lipopolysaccharide [2,3]. Although the bacterium induces a wide spectrum of human infections, including those of wounds, the eye, the gastrointestinal and the urinary tracts, it is most noted for catheter-associated urinary tract infections (CAUTIs). This opportunistic pathogen causes up to 44% of CAUTIs [2]. The catheterization period is recognized as a crucial risk factor of CAUTIs development. Almost all long-term catheterized (>28 days) patients develop a CAUTI, whereas in short-term (<7 days) catheterized patients, only 10–50% develop an infection. Furthermore, *P. mirabilis*-mediated CAUTIs are typically recurrent and complicated due to the formation of bladder or renal stones, acute pyelonephritis or kidney damage, leading to bacteremia, sepsis and even to death [3].

Urinary tract infections (including those involving *P. mirabilis*) are initiated by bacterial adhesion to an epithelial surface or a urinary catheter [4]. Among cell-surface structures mediating initial bacterial adhesion, an especially important role is played by lipopolysaccharide, bacterial nanofibers and fimbriae. Lipopolysaccharide, the major component of outer membranes of Gram-negative cells, consists of three regions: the most external O-polysaccharide (OPS), the core region and lipid A. The O-polysaccharide chains of *Proteus* LPS seem to have the most significant influence on bacterial initial surface adhesion. The serotype-specific antigen has much longer polysaccharides than the common antigen. It was assumed that 1000 hydrogen bonds between the common antigen chain and the surface are sufficient for firm cell attachment [5]. On the other hand, it was documented that a reduction in the length of the core region of LPS increased the ability of bacterial cells to adhere [6], indicating the significance of core LPS for the cell adhesion process. The best-known proteinous adhesins of Gram-negative bacteria are fimbriae. *P. mirabilis* produces mannose-resistant *Proteus*-like (MR/P) fimbriae and *P. mirabilis*-like fimbriae (PMFs), which facilitate bladder and kidney colonization, as well as non-agglutinating fimbriae (NAFs), which are important for uroepithelial cell colonization [4]. However, the significance of the rest of the 17 identified types of *P. mirabilis* fimbriae in initiating the adhesion process is still undescribed [7]. On the other hand, autotransporters (ATs) are the most frequently characterized surface proteins as non-fimbrial adhesins specific to Gram-negative pathogenic bacteria [5]. Alamuri et al. [8] discovered protein PMI2122 belonging to the AT-2 subfamily of autotransporters, playing a crucial role in the adhesion of *P. mirabilis* strain HI4320.

Cell appendages such as polysaccharide chains and proteinous nanofibers have an indisputable significance for bridging between cells and various surfaces. However, it is worth mentioning the biochemical and physical properties of membrane lipids, which can modulate the adherence process by creating focal adhesion points [9]. The influence of the fatty acids and phospholipids composition on the bacteria adherence ability has been documented [10,11,12]. This impact may be direct, as in the case of quantitative changes of particular lipid molecules which support or inhibit membrane adhesion, or indirect when altered membrane composition affects cell properties such as cell-surface hydrophobicity (CSH) and cell-surface charge (CSC) [13].

CSH is dependent on both the cultivation conditions (aeration, temperature, medium composition) and the growth phase of bacterial culture, and it is an individual feature of a species or even a strain [14]. Hydrophobicity is considered to be a factor which greatly affects microbial adhesion as well as cell self-agglutination during biofilm formation [13]. Besides, electric interactions of a double layer are thought to be a crucial factor in initial microbial adhesion. CSC of Gram-negative bacteria is mainly determined by negatively charged polysaccharides (containing uronic acids, sulfate and phosphate groups), proteins and lipids (especially by phosphatidylglycerols) of the outer membrane [15]. Moreover, it is suspected that changes in the inner membrane potential may also be important for the development of microbial biofilm [16,17], but this hypothesis is new and requires verification.

On the background of other uropathogens, the lipidome of *Proteus* sp. appears to be the least known. The focus of current research seems to be directed at *E. coli*-mediated infections, while studies on *P. mirabilis* lipids were published mostly in the 1970s, 1980s and 1990s. Although literature data provide information about fatty acids [18,19,20,21] and phospholipids composition [22] of *P. mirabilis* strains, their effect on microbial adhesion, a key factor for initial pathogenesis, has not been discovered. Except for the residual data about fatty acids and membrane hydrophobicity [23], there is no information on the connection of membrane lipids with both hydrophobic properties and membrane potential of *P. mirabilis.*

The present paper was designed to expand the knowledge on the mechanism of *P. mirabilis* adhesion to the catheter surface through analyses at the lipidome level. For this purpose, three isolates of different origin and exhibiting different adhesion abilities were investigated in search of fatty acids and phospholipids, which could indirectly affect physicochemical cell properties such as a hydrophobicity, the inner membrane potential and a generalized polarization, which in turn are assumed to be significant for the colonization of a catheter surface by *P. mirabilis*. In this paper, gas chromatography coupled with mass spectrometry (GC-MS), high-performance liquid chromatography coupled with tandem mass spectrometry (LC-MS/MS) and fluorescent techniques were combined to delve into the mechanism of *P. mirabilis*-mediated colonization of urological catheters.

## 2. Materials and Methods

### 2.1. Microorganisms and Growth Conditions

The bacteria belonging to *Proteus mirabilis* species were examined in this study. The strains named as 1984 and C12, which were obtained from the Department of Biology of Bacteria (University of Lodz, Lodz, Poland), came from patients hospitalized in Lodz. *P. mirabilis* 1984 was isolated from urine, while *P. mirabilis* C12 was isolated from a urinary catheter. The reference strain *P. mirabilis* ATCC 29906 was purchased from the American Type Culture Collection (Manassas, VA, USA).

Bacteria were stored as frozen stocks in the presence of DMSO (−80 °C). The colonies grown on an agar nutrient plate with 0.1% phenol (swarming growth inhibitor) were used to inoculate Tryptic Soy Broth (TSB, BioMaxima S.A., Lublin, Poland) medium in 100 mL Erlenmeyer flasks. The cultivation was carried out at 37 °C with shaking at 105 rpm for 24 h. Planktonic cells were collected every 2 h (for growth, adhesion and hydrophobicity studies) and after 6 and 24 h incubations (for lipidomic and fluorescent studies).

Adherent cells were obtained in the following way. The silicone-coated catheters (14Fr/ch 30 mL/cc 4.7 mm, Cezetel-Poznan, Poznan, Poland) were divided into 0.5 cm pieces for adhesion and fluorescent tests and 1.0 cm pieces for other tests. The pieces of catheters were placed into 100 mL Erlenmeyer flasks containing 24 h cultures of *P. mirabilis* diluted 10-fold in TSB medium. The cultivation was carried out at 37 °C with shaking at 105 rpm for another 24 h. Adherent cells were collected every 2 h (for growth, adhesion and hydrophobicity studies) and after 6 and 24 h incubations (for lipidomic and fluorescent studies).

Studies were performed for 6 and 24 h *P. mirabilis* cells to capture changes in the bacterial lipidome during the early and mature phases of biofilm development. After 6 h of incubation, the urological catheter was thinly and reversibly colonized by *P. mirabilis* cells, which was assumed to be the early phase, while after 24 h of incubation, the silicone surface was densely and irreversibly colonized by bacterial cells, which was assumed to be the late stage.

### 2.2. Bacterial Growth

Planktonic cells of *P. mirabilis* strains were obtained according to Section 2.1. The optical density (OD) of 1 mL suspensions was measured spectrophotometrically (Ultrospec 2000, Pharmacia Biotech, Uppsala, Sweden) at λ = 550 nm. These data were used for drawing a growth curve. In addition, 1 mL suspensions were centrifuged for 15 min at 12,000 rpm and dried at 105 °C to constant weight. The maximum specific growth rate (μ_max_) was calculated in accordance with the formula μ_max_ = [(lnX_2_ − lnX_1_)/(t_2_ − t_1_)], where X_2_ is the biomass concentration at time t_2_ and analogously for X_1_.

### 2.3. Estimation of Cell-Surface Hydrophobicity (CSH)

Planktonic cells of *P. mirabilis* ATCC 29906, 1984 and C12 strains were prepared according to Section 2.1. The hydrophobic cell properties were estimated according to Rosenberg et al.’s [24] method with some modifications. First, bacterial cultures were transferred into 50 mL Falcon tubes, centrifuged for 15 min at 4500 rpm and washed twice with PUM buffer (K_2_HPO_4_ × 3H_2_O (22.2 g), KH_2_PO_4_ (7.26 g), CH_4_N_2_O (1.8 g), MgSO_4_ × 7H_2_O (0.2 g), distilled water up to 1000 mL, pH 7.1). The washed cells were suspended in PUM buffer, obtaining 1 × 10^9^ CFU mL^−1^ (A_550_ was about 1.0). Then, 1.2 mL of standardized suspension was transferred into a 2 mL Eppendorf tube. Next, 20 µL of *p*-xylene (Avantor, Gliwice, Poland) was added and the mixture was preincubated for 10 min at 37 °C. After preincubation, it was intensively vortexed for 3 min and left for 30 min for phase separation. Subsequently, the absorbance of the lower phase was measured again. CSH, calculated in accordance with the formula CSH = [(a − b)/a] × 100% (where a is an initial absorbance and b is an absorbance after phase separation), was reported as the percentage of total cells partitioned into the hydrocarbon. *P. mirabilis* strains that had a CSH value ≥ 50% were considered as hydrophobic.

### 2.4. Study on the Cell Adhesion Ability

Adherent cells of the studied bacteria were obtained according to Section 2.1. Control samples were pieces of a catheter incubated in sterile TSB medium. The crystal violet test was useless due to the fact that it resulted in the permanent coloration of the catheter surface, therefore it was decided to determine the number of cells attached to the catheter surface using a MTT tetrazolium reduction assay, assuming that dead cells represent a small percentage. A procedure was conducted according to Grela et al.’s [25] method with a few modifications. The catheter pieces were transferred from liquid cultures into a glass dish filled with sterile distilled water to remove planktonic cells. Next, the catheter pieces were placed in a 96-well plate containing 150 µL of TSB medium and 15 µL of MTT (3-(4,5-dimethylthiazol-2-yl)-2,5-diphenyltetrazolium bromide (Merck, Darmstadt, Germany), 5 mg mL^−1^ in PBS buffer). The samples were incubated in a moist chamber for 30 min at 37 °C. After incubation, the catheter pieces were transferred to new wells. Formazan crystals accumulated inside adherent cells were dissolved in 175 µL of DMSO (Merck, Darmstadt, Germany) and glycine buffer (Merck, Darmstadt, Germany) mixture (6:1 *v*/*v*). A_550_ was measured using a microplate reader (Multiskan EX, LabSystem, Cracow, Poland).

### 2.5. Isolation of Cell Lipids

Planktonic and adherent cells were obtained according to Section 2.1. Lipids were extracted from the whole cell homogenate without fractionation, i.e., without separating the outer and the inner membranes.

The planktonic cells were transferred into 50 mL Falcon tubes, centrifuged for 15 min at 4500 rpm and washed twice with sterile distilled water, while the catheter pieces were firstly transferred to a glass dish with sterile distilled water to remove planktonic cells. Next, the catheter pieces were placed into 50 mL Falcon tubes containing 20 mL of sterile distilled water. The content of the tubes was sonicated in an ultrasonic cleaner for 20 min at room temperature and subsequently vortexed for 2 min. The catheter pieces devoid of bacteria were removed from the tubes and centrifuged for 15 min at 4500 rpm. The planktonic and adherent cells suspended in 1 mL of sterile distilled water were placed into a 2 mL Eppendorf tube and centrifuged for 15 min at 12,000. The biomass prepared in this way was used for the isolation of cell lipids.

Bacterial phospholipids were isolated from whole cells using the method described by Bligh and Dyer [26]. The applied procedure was more appropriate for extraction efficiency, obtaining a variety of lipids and repeatability of results, than the methods developed by Stolarek et al. [27] and Matyash et al. [28] (data not shown). Firstly, 1 mL of methanol (Chempur, Piekary Slaskie, Poland) and glass beads was added to the bacterial biomass. A homogenization process was conducted 3 times for 20 s at 20 m s^−1^ using a ball mill (FastPrep-24, MP-Biomedicals, Santa Ana, CA, USA). The homogenate was placed into a 15 mL Falcon tube with 1 mL of chloroform and 800 µL of distilled water. The mixture was vortexed for 3 min and left for 5 min for phase separation. Next, the bottom layer was collected and evaporated to dryness. Lipids were dissolved in ultrapure methanol (J.T. Baker Chemical Company, Deventer, The Netherlands) and stored at −80 °C.

### 2.6. Preparation of Bacterial Fatty Acid Methyl Esters (FAME)

All membrane phospholipids of the *P. mirabilis* ATCC 29906, 1984 and C12 strains, obtained according to Section 2.5, were methanolysed according to Ichihara and Fukubayashi’s [29] procedure. In short, the lipids dissolved in 1.5 mL of methanol (Chempur, Piekary Slaskie, Poland) were transferred into a Pyrex tube. Next, 200 µL of toluene and 300 µL of 8% HCl were added. The mixture was vortexed for 3 min and incubated for 18 h at 45 °C. After cooling the mixture at room temperature, 1 mL of ultrapure hexane (J.T. Baker Chemical Company, Deventer, The Netherlands) and 1 mL of deionized water were added. The pyrex content was vortexed for 3 min. Finally, 300 µL of the upper layer was collected and transferred to a chromatographic vial.

### 2.7. Qualitative Analysis of Fatty Acids by GC-MS Technique

FAMEs prepared according to Section 2.6 were measured using an Agilent Model 7890 gas chromatograph equipped with a 5975C mass detector (Santa Clara, CA, USA). Then, 1.6 µL of the sample was injected onto a capillary column HP 5 MS methyl polysiloxane (30 m × 0.25 mm i.d. × 0.25 mm ft) with the helium flow of 1 mL min^−1^ and injection port at 250 °C. The temperature of the column was maintained for 3 min at 60 °C, then increased to 212 °C at the rate of 6 °C min^−1^, followed by an increase to 245 °C at the rate of 2 °C min^−1^, and finally, to 280 °C at the rate of 20 °C min^−1^, at which it was held for 10 min. Bacterial fatty acids of all *P. mirabilis* membranes were identified by comparison with reference standards (Matreya LLC, State College, PA, USA) or by their mass spectra. The exemplary mass spectra of palmitoleic acid (*cis* C16:1) and stearic acid (C18:0) are shown in Supplementary Figure S1. Quantitative results read from the standard curve (µg mL^−1^) have been converted into relative values (%), where the sum of total isolated fatty acids was 100%.

### 2.8. Quantitation of Phospholipids by LC-MS/MS Method

Phospholipids of studied bacteria were isolated according to Section 2.5. The polar lipids were measured according to the method of Bernat et al. [30]. An Agilent 1200 HPLC system (Santa Clara, CA, USA) and a 4500 Q-TRAP mass spectrometer (Sciex, Framingham, MA, USA) equipped with an ESI source working in the negative mode were applied. Then, 10 μL of the lipid extract was injected onto a Kinetex C18 column (50 mm × 2.1 mm, particle size: 5 μm; Phenomenex, Torrance, CA, USA) and heated to 40 °C, with the flow rate of 500 µL min^−1^. Water (A) and methanol (B) (J.T. Baker Chemical Company, Deventer, The Netherlands) were applied as a mobile phase, with both containing 5 mM ammonium formate. The solvent gradient was initiated at 70% B and, after 0.25 min, increased to 95% B for 1 min; then, it was maintained at 95% B for 7 min before returning to the initial solvent composition over 2 min. Based on the product ion and precursor ion analysis of head groups and fatty acyls, a comprehensive list of multiple reaction monitoring (MRM) transitions was generated. All bacterial phospholipids were identified based on reference standards (Avanti Polar Lipids, Alabaster, AL, USA) or by their mass spectra. The data analysis was conducted with the Analyst™ v1.6.2 software (Sciex, Framingham, MA, USA).

The exemplary mass spectra of PE 32:0 and PG 33:1 are shown in Supplementary Figure S2. Quantitative results read from the standard curve (µg mL^−1^) have been converted into relative values (%), where the sum of total isolated fatty acids was 100%. The Double Bond Index (DBI) describing the unsaturation of phospholipid fatty acids was calculated in accordance with the formula of Su et al. [31].

### 2.9. Fluorescent Probe Investigations

Planktonic and adherent cells were obtained according to Section 2.1. The evaluation of the inner membrane potential was conducted using bis-(1,3-dibutylbarbituric acid)trimethine oxonol (DiBAC_4_(3), Merck, Darmstadt, Germany) according to the modified method by Stolarek et al. [27]. In order to determine a generalized polarization value, the bacterial cells were labeled with 6-dodecanoyl-N,N-dimethyl-2-naphthylamine (Laurdan, Merck, Darmstadt, Germany) according to the procedure described by Stolarek et al. [27], while measurements of GP were carried out based on the Hofstetter method [32].

The planktonic and adherent cells were washed out analogously as at the beginning of lipid isolation (the second paragraph in Section 2.5). The mixture contained the cells, 1 mL of PBS buffer and 2 µL of DiBAC_4_ (3) (1 mg mL^−1^ in EtOH) or 12 µL of Laurdan (0.5 mM in EtOH). The samples with DiBAC_4_ (3) were incubated for 5 min in the dark at room temperature, while the samples with Laurdan were incubated for 45 min at 37 °C. The fluorophore residues were removed by centrifuge for 15 min at 12,000 rpm and 2-fold washed with PBS buffer.

The prepared samples were placed into a 96-well plate. The measurements of fluorescence intensity values of DiBAC_4_ (3)-labeled cells were performed using a FLUOstar Omega spectrofluorometer (BMG Labtech, Ortenberg, Germany) with the following parameters: λex = 485 − 12 nm, λem = 520 nm, gain = 1000, target = 70%. The results are shown as a fluorescence intensity unit (with regard to OD_550_ before labeling). The fluorescence intensity values of Laurdan-labeled cells were measured with use of a Cary Eclipse Fluorescence Spectrophotometer (Agilent, Santa Clara, CA, USA) with the following parameters: λex = 360 nm, λem_1_ = 440 nm, λem_2_ = 490 nm. GP values were calculated in accordance with the following formula: GP = (I_440_ − I_490_)/(I_440_ + I_490_).

### 2.10. Statistical Analyses

The experimental data represent the means of at least three independent experiments. An average standard deviation (±SD) was calculated. The one-way ANOVA, a Fisher’s test, a Spearman’s correlation and PCA analysis were performed. Values were considered significant when *p* ≤ 0.05. The analyses were performed using the Excel 2016 (Microsoft Corporation, Redmond, WA, USA) and MarkerView™ Software 1.2.1 (Sciex, Framingham, MA, USA).

## 3. Results

### 3.1. Growth, Cell Hydrophobicity and Adhesion of the Pathogens to a Catheter Surface

**Figure 1:**
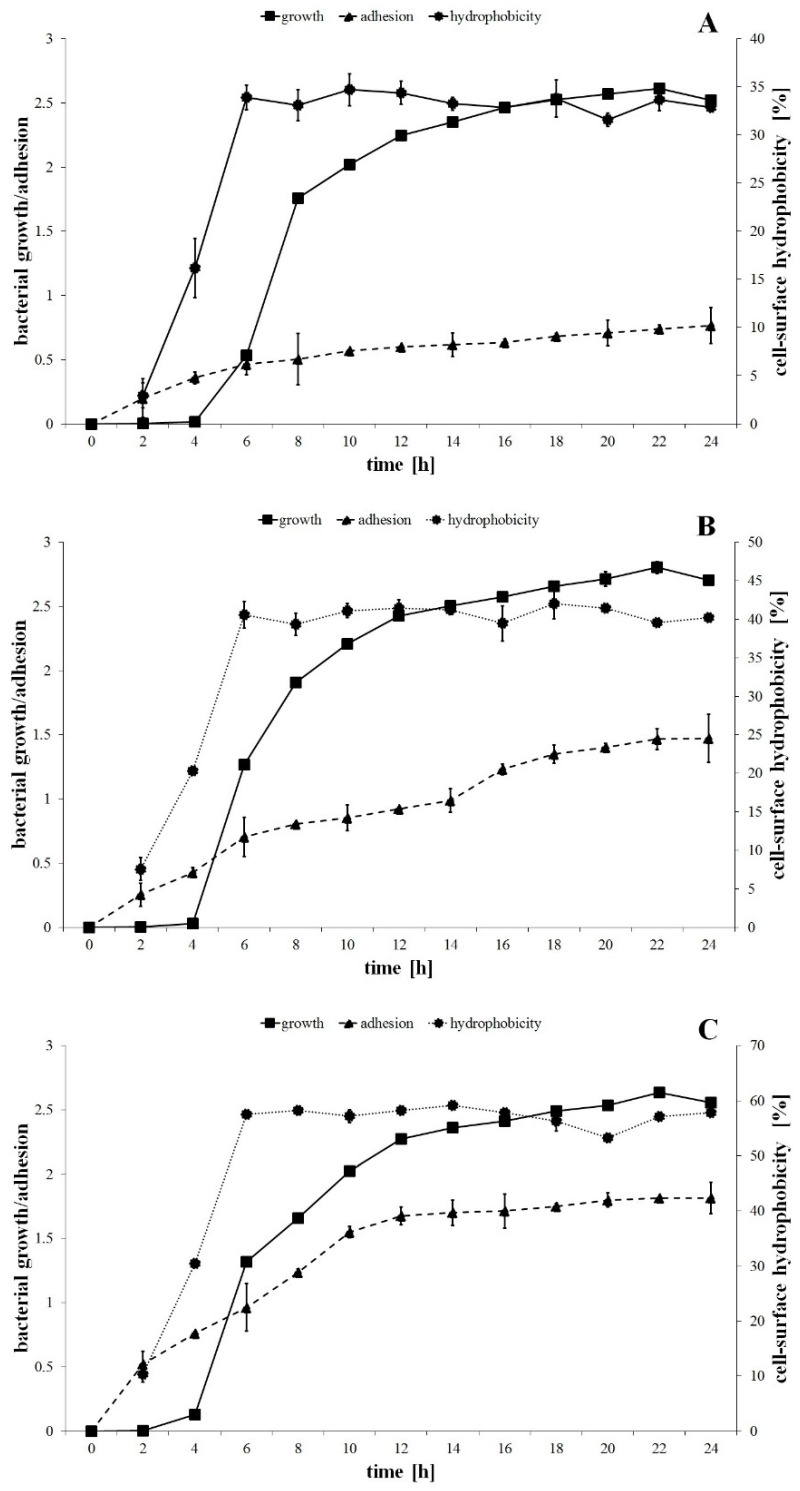
The bacterial growth, adhesion to the catheter and cell-surface hydrophobic properties of the *P. mirabilis* ATCC 29906 (**A**), 1984 (**B**) and C12 (**C**) strains.

The growth curves of the *P. mirabilis* strains are shown in Figure 1. One-way ANOVA showed no significant differences between the growth kinetics of the tested bacteria. However, the maximum specific growth rates (µ_max_) calculated for the biomass increment between 4 and 6 h of incubation were respectively 1.75, 1.89 and 1.16 h^−1^ for *P. mirabilis* ATCC 29906, 1984 and C12 strains.

The studied strains were differentiated in terms of cell hydrophobic properties. The cell-surface hydrophobicity values were equal to 3%, 7.5% and 10.3% for 2 h cells of the *P. mirabilis* ATCC 29906, 1984 and C12 strains, respectively (Figure 1). During the exponential growth phase, the percentage of cell hydrophobicity increased respectively to 33.9%, 40.6% and 57.5%, while during the stationary growth phase it remained unchanged. The Fisher′s test showed significant differences (*p* ≤ 0.05) between the *P. mirabilis* ATCC 29906 and the C12 strains (Figure 1A,C), pointing to the first as the most hydrophilic and to the second as the most hydrophobic.

The adhesion process of *P. mirabilis* strains progressed over time (Figure 1). One-way ANOVA showed that the adhesion kinetic of the tested bacteria differed statistically (*p* ≤ 0.01). In the first 6 h of the experiment, the catheter surface was colonized the fastest by the cells of the C12 strain (OD_550_ = 0.963), followed by the 1984 (OD_550_ = 0.703) and the ATCC 29906 (OD_550_ = 0.466) strains, respectively. After 24 h of incubation, the quantity of the hydrophobic *P. mirabilis* C12 cells adhering to the catheter surface was more than 2-fold higher than that of the hydrophilic *P. mirabilis* ATCC 29906 cells.

### 3.2. Fatty Acid Composition

The identification of fatty acids methyl esters located in both the outer as well as the inner membranes of the *P. mirabilis* was conducted using the GC-MS technique. An analysis of the mass spectra showed the presence of 16 straight-chain fatty acids and 5 branched-chain fatty acids (FAs) in the bacterial membranes. The acyl chains were composed of 10 to 20 carbon atoms. Most of the identified fatty acids were saturated. Only palmitoleic acid (C16:1), oleic acid (C18:1) and linoleic acid (C18:2) contained double bonds. *Trans* fatty acids were not found. On the other hand, 5 saturated, unbranched FAs with attached hydroxyl groups in the α or β chain position (C10:0 2OH, C12:0 2OH, C12:0 3OH, C14:0 3OH, C16:0 2OH) were noted. Among the branched fatty acids, only one *iso*- form was found: 14-methylpentadeconatic acid (*iso* C16:0). *Anteiso* forms were not detected. Moreover, 2 alicyclic FAs were identified, i.e., *cis*-9,10-methylenehexadecanoic acid (*cy* C17:0), also known as 8-(*cis*-2-hexylcyclopropyl)octanoic acid, and *cis*-9,10-methyleneoctadecanoate (*cy* C19:0), also known as 8-(*cis*-2-octylcyclopropyl)octanoic acid.

**Figure 2:**
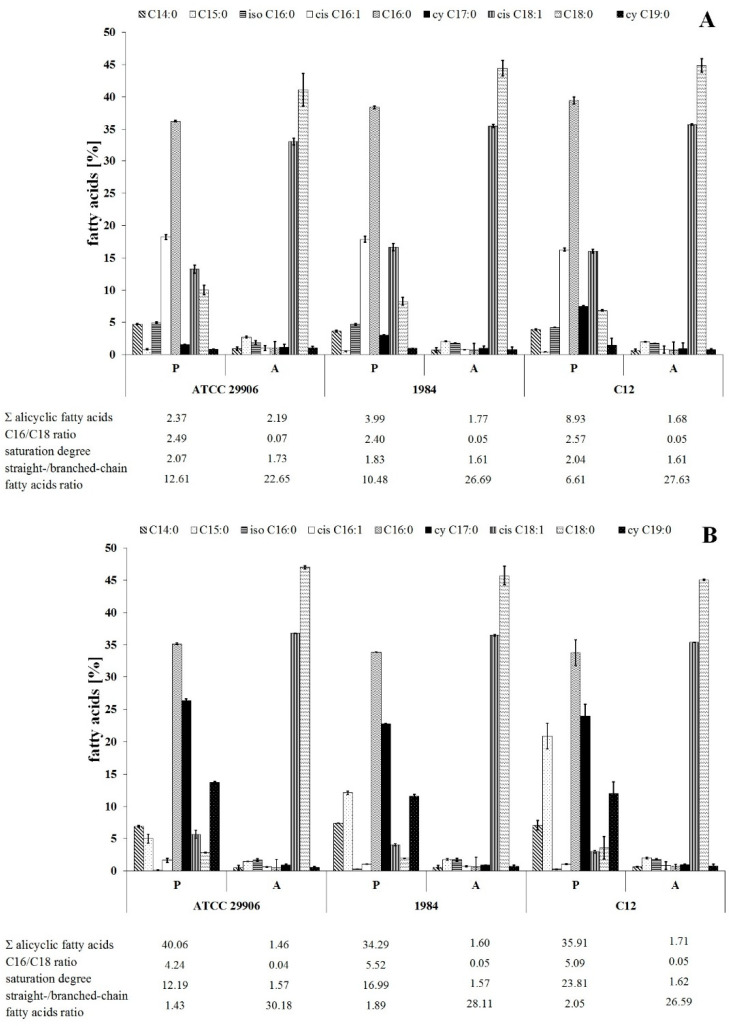
Relative abundances of fatty acids isolated from both the outer and the inner membranes of 6 h (**A**) and 24 h (**B**) planktonic (P) and adherent (A) cells of *P. mirabilis* ATCC 29906, 1984 and C12.

The relative amounts of dominant FAs identified in all of the *P. mirabilis* membranes are shown in Figure 2. The others, whose level was below 1%, have been omitted. The studied bacterial membranes were mainly composed of myristic acid (C14:0), pentadecylic acid (C15:0), palmitic acid (C16:0), palmitoleic acid (*cis* C16:1), *cy* C17:0, C18:1, stearic acid (C18:0) and *cy* C19:0, while the summed abundances of C16:0, *cis* C16:1, C18:0 and C18:1 were in the range from 40% to 85% of total fatty acids.

The present study was designed to compare the fatty acids profiles for adherent and planktonic cells of the *P. mirabilis* strains in search of FAs related to the phenomenon of cell adherence to a catheter surface. The adherent (A) and the planktonic (P) cells were different (*p* ≤ 0.01) in terms of their FA composition, however the distribution of fatty acids between the outer and the inner membranes was not determined. First of all, the A cells had higher contents of *cis* C18:1 and C18:0 as well as lower levels of *cis* C16:1, C16:0 and *cy* 19:0 than the P cells (Figure 2). Moreover, adherent cells were characterized by less content of acyclic fatty acids and longer, more unsaturated and less branched fatty acids than planktonic cells.

The dependences between the FA profile of the *P. mirabilis* adherent cells and their ability of catheter surface colonization were analyzed using a Spearman’s correlation. A strong negative relation was found between the C11:0 level and the ability of the tested bacteria to cover the catheter. Moreover, hydrophobic properties of the *P. mirabilis* ATCC 29906, 1984 and C12 planktonic cells were negatively correlated with *cis* C16:1 levels, as well as positively dependent on the saturation degree of fatty acids (correlation coefficients are presented in Supplementary Table S1).

### 3.3. Phospholipid Profile

**Figure 3:**
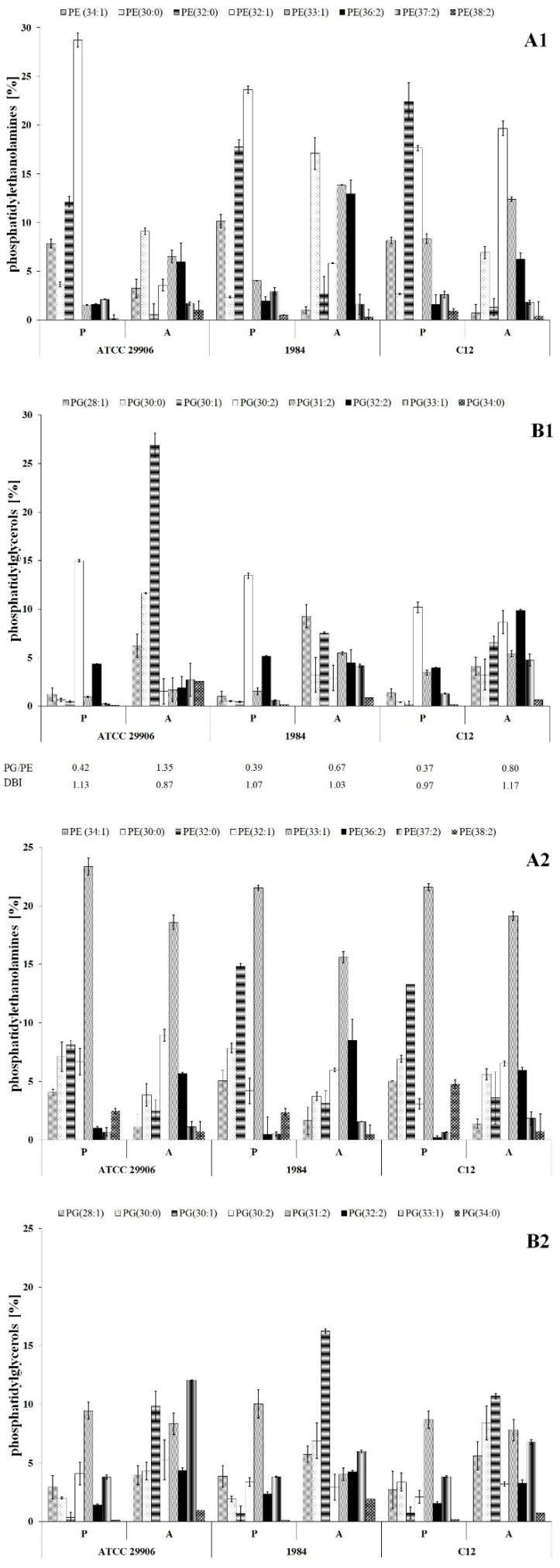
Relative abundances of phosphatidylethanolamines (**A1**,**A2**) and phosphatidylglycerols (**B1**,**B2**) isolated from both the outer and the inner membranes of 6 h (**A1**,**B1**) and 24 h (**A2**,**B2**) planktonic and adherent cells of *P. mirabilis* ATCC 29906, 1984 and C12.

Qualitative analysis of phospholipids performed using the LC-MS/MS technique showed phosphatidylethanolamines (PE) and phosphatidylglycerols (PG) as the main components of the *P. mirabilis* outer and inner membranes. Sixteen PE species and eleven PG species were identified, which represented respectively about 70% and 30% of the total phospholipids amount in planktonic cells (Figure 3). The acyl chains of PE consisted of 30 to 38 carbon atoms, while PG molecules had fatty acids containing 28 to 34 carbon atoms. The identified phospholipids contained saturated, mono-unsaturated and di-unsaturated fatty acids. Quantitatively, PE 32:1 followed by PE 32:0, PG 30:2 and PE 34:1 had the highest levels.

The present study was designed to compare the phospholipid profiles for adherent and planktonic cells of the *P. mirabilis* strains in search of PLs which can facilitate the catheter surface colonization. The adherent cells differed in their phospholipid profile from the planktonic cells (*p* ≤ 0.01), however the distribution of the lipids between the outer and the inner membranes was not determined. First of all, the A cells had significantly higher amounts of PE 33:1, PE 34:0, PG 28:1, PG 30:0, PG 30:1 and PG 33:1 than the P cells. On the other hand, PE 34:1, PE 32:0, PE 32:1, PG 30:2 and PG 32:2 quantities were significantly decreased in the adherent cells compared to the planktonic cells. Apart from 6 h cells of the 1984 strain, in the other cases, the A cells had higher ratios of phosphatidylglycerols to phosphatidylethanolamines than the P cells. Contrary to PG/PE ratios, DBI values indicating the degree of fatty acids unsaturation remained constant (~1.0) for both planktonic and adherent cells of *P. mirabilis* ATCC 29906, 1984 and C12 strains.

**Figure 4:**
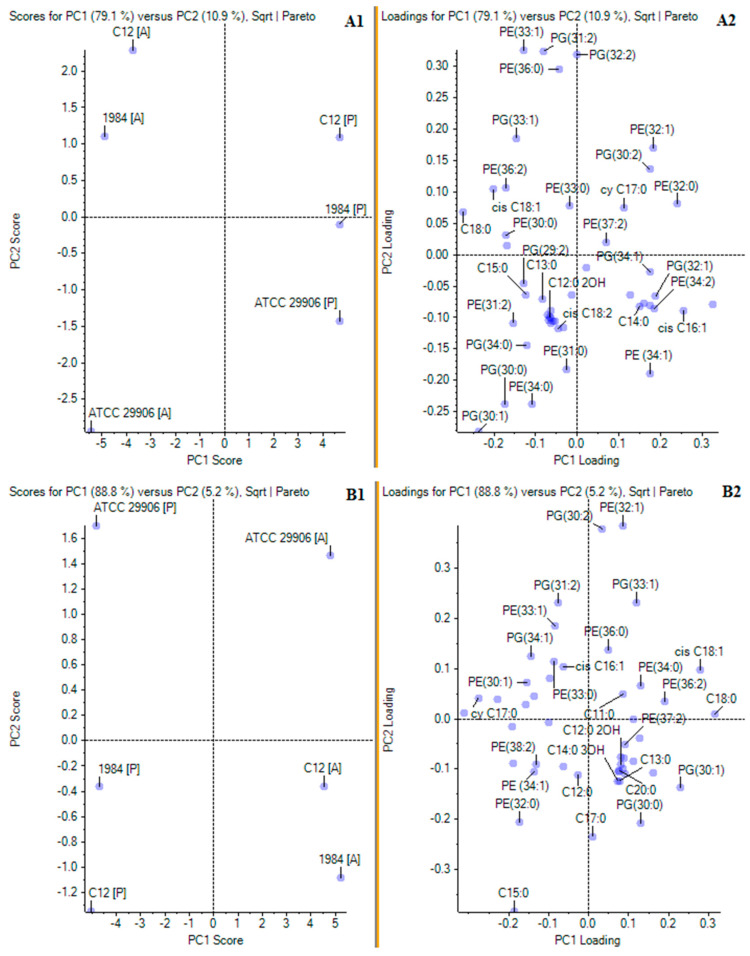
Principal component analyses of FA and PL profiles of planktonic and adherent cells of the studied *P. mirabilis* strains. Panels (**A1**,**A2**) and (**B1**,**B2**) present PCA for respectively 6 and 24 h *P. mirabilis* ATCC 29906, 1984 and C12 cells. Panels marked 1 (**A1**,**B1**) show PC1 and PC2 scores, while panels marked 2 (**A2**,**B2**) show PC1 and PC2 loadings.

In order to confirm the main differences in the profiles of phospholipids and fatty acids incorporated in the planktonic and the adherent cells of the *P. mirabilis* strains, a principal component analysis (PCA) was applied. The matrix loadings were orthogonally rotated, and the first four principal components were retained based on the examination of the scree plot of the eigenvalues. In the PCA of the relative levels of FAs and PLs, the two principal components explained 79.1% and 10.9% of the overall variances for 6 h cells (Figure 4A1), as well as 88.8% and 5.2% of the overall variances for 24 h cells (Figure 4B1). The location of the P and the A cells in various halves of the coordinate system proved the differences in the planktonic and the adherent *P. mirabilis* cells in terms of cell composition (without division to lipids of the outer and the inner membrane). Firstly, PCA of the results from 6 h of incubation revealed that the cells of the *P. mirabilis* C12 and 1984 strains were similar in terms of lipid composition. The A cells of both strains were found in the first quarter, whereas the P cells of both strains were located in the second quarter. On the other hand, the P and A cells of the *P. mirabilis* ATCC 29906 strain were located separately, respectively in the third and in the fourth quarter, being completely distinct from the rest (Figure 4A1). A very similar distribution of the samples was obtained for the 24 h cells with the difference that the examined cells were relocated between different quarters (Figure 4B1). According to the results of the principal component analysis, the molecules whose levels separated the 6 h C12 and ATCC 29906 adherent cells (respectively as highly and weakly adherent strains) were PE 33:1, PE 36:0, PG 31:2, PG 33:1, PG 34:0, C13:0 and C15:0. The 24 h C12 and ATCC 29906 adherent cells were differentiated by the levels of PE 32:1, PG 30:0, PG 33:1 and C11:0. On the other hand, the lipids whose amounts distinguished the planktonic cells from the adherent cells of the *P. mirabilis* C12 (as the most adherent and hydrophobic strain) were PE 32:0, PE 33:1, PG 32:2, PG 33:1 and *cy* 17:0. The 24 h adherent and planktonic cells of the C12 strain were differentiated by the quantities of PE 34:1, PE 32:0, PE 38:2, PG 30:0, PG 30:1 and C15:0.

The relations between the PL profile of the *P. mirabilis* adherent cells and their capacity to adhere to urological catheter surfaces were analyzed with the use of a Spearman’s correlation. A positive dependence between the ability of catheter colonization and the PE 37:2 level, as well as a negative correlation between the ability of adhesion and both the sum of unsaturated PGs and PG/PE ratios, were noticed. A Spearman’s correlation also showed that the hydrophobicity of *P. mirabilis* cells was positively dependent on PE 32:0, PE 33:0, PE 38:2, PG 33:1 and PG 34:0 levels, as well as negatively related to PE 30:1, PE 32:1 and PG 30:2 contents, the sum of unsaturated PGs, PG/PE ratios and DBI values (correlation coefficients are presented in Supplementary Table S1).

### 3.4. Inner Membrane Potential

**Figure 5:**
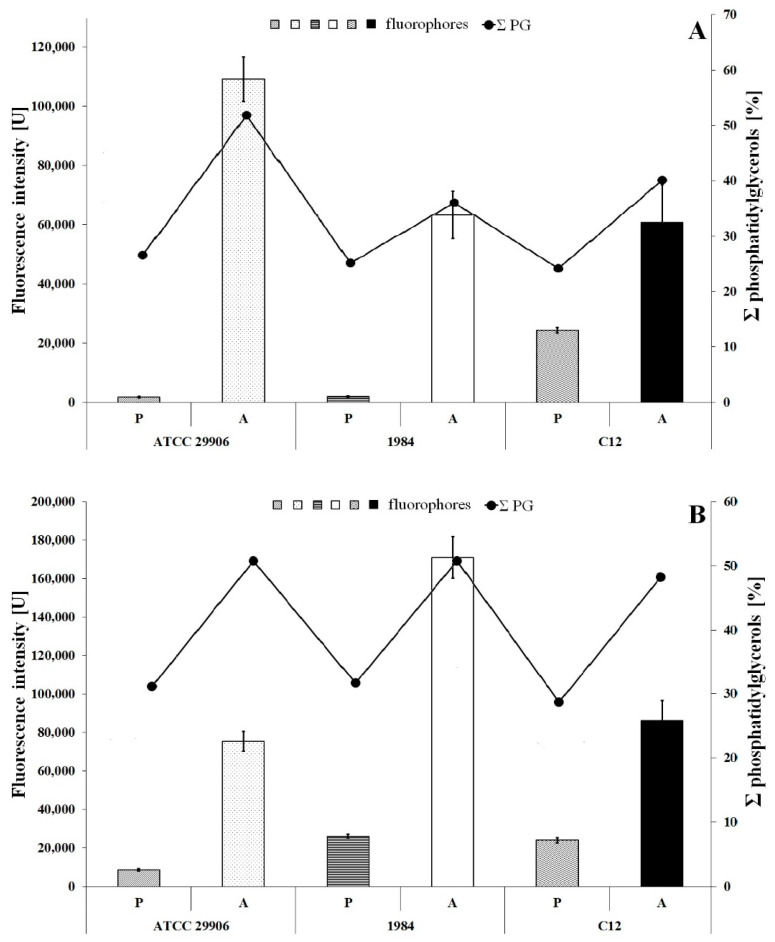
Fluorescence intensity of 6 h (**A**) and 24 h (**B**) planktonic and adherent cells of the *P. mirabilis* ATCC 29906, 1984 and C12 labeled with DiBAC_4_ (3) (a bar graph) and the summed content of phosphatidylglycerols in the tested bacterial cells (a line graph).

The inner membrane potential of the studied *P. mirabilis* cells was measured fluorescently using the anionic fluorophore DiBAC_4_ (3). The obtained results are presented in Figure 5. The fluorescence measurements of the labeled 6 h planktonic cells were 1817 U, 2040 U and 24447 U for *P. mirabilis* ATCC 29906, 1984 and C12 strains respectively, while fluorescence intensities of the 24 h planktonic cells were 8646 U, 26123 U and 24056 U for *P. mirabilis* ATCC 29906, 1984 and C12 strains, respectively. The values obtained for all A cells were statistically significantly (*p* ≤ 0.05) lower than those of the P cells. The slightest differences were observed for the *P. mirabilis* C12 strain. DiBAC_4_ (3) emission was up to 3.5-fold lower for the A cells compared to the P cells. However, fluorescence intensities of the labeled adherent cells were even up to 30- and 60-fold lower in *P. mirabilis* 1984 and *P. mirabilis* ATCC 29906 inner membranes respectively, compared to those for the P cells. Due to the anionic character of DiBAC_4_ (3), it is able to penetrate depolarized cells. An increase in the fluorophore emission corresponds to a less negative charge inside the cell. Therefore, the obtained results point to the inner membrane’s depolarization of the *P. mirabilis* cells adhering to catheters.

An inner membrane polarization is related to the presence of charged membrane components, and therefore Figure 5 additionally shows the content of phosphatidylglycerols in the *P. mirabilis* cells. An increase in the PG amount in the bacterial membrane is not accompanied by a decline in fluorescence intensity of the cytoplasmic membrane. This result indirectly eliminates the influence of PGs level on the value of inner membrane potential.

### 3.5. Fluidity of P. mirabilis Membranes

**Figure 6:**
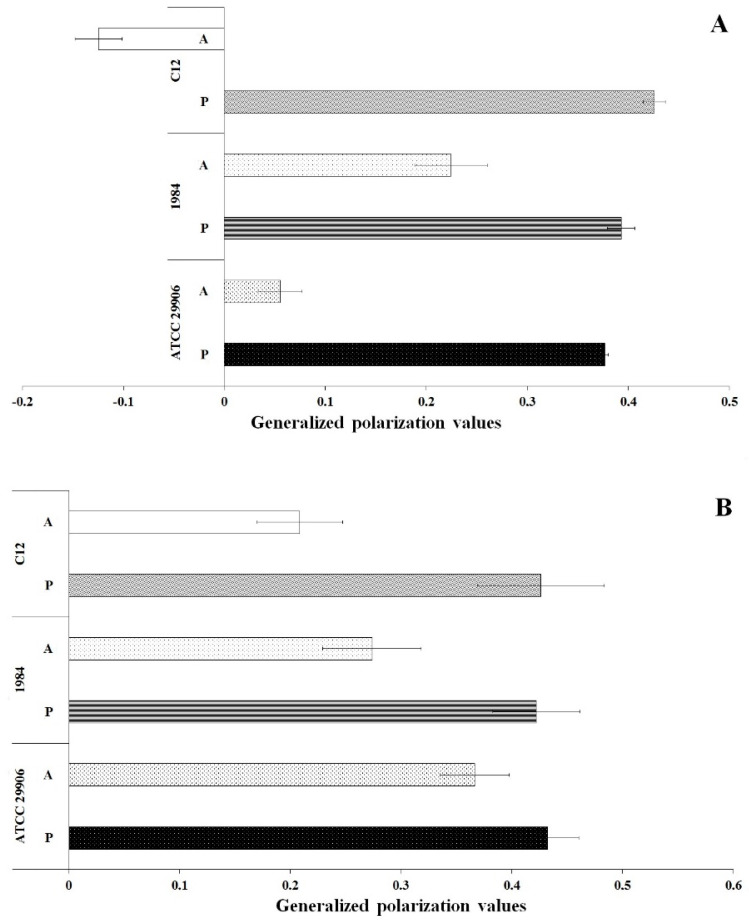
General polarization values of 6 h (**A**) and 24 h (**B**) planktonic and adherent cells of the *P. mirabilis* ATCC 29906, 1984 and C12.

Membrane fluidity was investigated through generalized polarization (GP) measurements of the bacterial cells labeled with Laurdan. The results are shown in Figure 6. In general, the GP values for all samples were in the range from −0.12 to 0.43. Significantly lower Laurdan GP values (*p* ≤ 0.05) were reported for the *P. mirabilis* cells attached to the catheter surface than for its planktonic cells. These results indicated lower packing density of membrane components of the A cells compared to tightly packed membranes of the P cells. Comparing GP values obtained for the planktonic and the adherent cells, the greatest differences were observed for 6 h cells of the highly adherent and hydrophobic strain, which is *P. mirabilis* C12. Generalized polarization of Laurdan was estimated at 0.43 for the P cells and at −0.12 for the A cells.

## 4. Discussion

Fimbriae are considered as the main surface structures mediating initial bacterial adhesion. *P. mirabilis* represents a particular case in which various types of fimbriae can be expressed simultaneously by the same cell. An arrangement of fimbriae on the *P. mirabilis* ATCC 29906, 1984 and C12 strains and their importance for adhesion have not been investigated so far. However, studies demonstrating fimbriae-mediated adhesion of other *P. mirabilis* strains to urinary tract tissues and blood cells have been reported by some authors. Participation of MR/P fimbriae in the *P. mirabilis* adhesion to T24 cells as well as the role of PMF fimbriae in the adherence to bladder and kidney tissues have been confirmed genetically with the use of mutants lacking the genes encoding MR/P and PMF fimbriae [33,34]. Jiang et al. recently published data on MrpH, a new class of metal-binding adhesin which requires zinc to mediate adhesion to erythrocytes [35]. A fimbriae could also be involved in bacterial adhesion to an abiotic surface, such as a urological catheter. Both MR/P and MR/K fimbriae are associated with the adhesion of *P. mirabilis* strains to the catheter material [36]. On the other hand, Scavone et al. [37] analyzed the role of MR/P, PMF, ATF (ambient temperature fimbriae) and UCA (uroepithelial cell adhesion) fimbriae of *P. mirabilis* in the adherence to the catheter surface, and they found that some mutants were less adhesive compared with the wild-type (MR/P and ATF), while others were more adhesive (UCA and PMF). The authors concluded that *P. mirabilis* fimbriae have distinguishable roles in the generation of biofilms, particularly in association with catheters. Non-fimbrial adhesins, which are a growing class of outer-membrane proteins (OMPs), also play a role in microbial adherence. A cyclic AMP receptor protein (Crp), as a major transcriptional regulator in bacteria, is necessary for kidney colonization [38], while PMI2122, a trimeric autotransporter with “adhesin-like” properties, is required for the *P. mirabilis* ability to adhere to HEK293 cell monolayers [8]. On the other hand, Clapham et al. [39] and Sin-Sien Jiang et al. [40] paid attention to inner-core LPS biosynthetic protein and UDP-glucuronic decarboxylase, whose inactivation resulted in the modification of the LPS structure, thus limiting the *P. mirabilis*-mediated adhesion.

It appears that the role of the above cell surface structures in supporting the initial adhesion of *P. mirabilis* cells is better known and described than smaller cell-building molecules such as lipids, which could be associated with the process. Therefore, we concentrated our research on phospholipids and fatty acids whose modifications influence the physicochemical properties of cells, which could be necessary for the attachment of the bacteria to the catheter surface.

Cell-surface hydrophobicity has been reported in a variety of potentially pathogenic bacteria, including *Legionella pneumophila* [41], *Acinetobacter baumannii* [42], *Serratia marcescens* [43], *Salmonella* sp. [44] and *Escherichia coli* [45], which suggests that CSH is a virulence attribute. Peerbooms et al. [46] investigated 20 strains of *P. mirabilis* which proved to differ in CSH, and 18 of them were hydrophobic with the affinity values for dextrane from 57% to 99%. On the other hand, the research of Mitsuyama et al. [47] and Czerwonka et al. [48] showed that all of the 5 examined *P. mirabilis* strains were hydrophilic because the degree of their hydrophobicity was found to be lower than 34%. Moreover, other *Proteus* species were also characterized by hydrophilic cell properties. Bartodziejska et al. [49] identified 22 out of the 25 tested *P. vulgaris* strains and 18 out of the 19 tested *P. penneri* strains as hydrophilic. As mentioned before, growth conditions and even strain features have significance for CSH. However, the knowledge of the cell-surface properties may predict the nature of the material to which the microorganism will adhere. According to the thermodynamic theory, bacteria with a hydrophobic cell surface prefer hydrophobic material surfaces, while bacteria with a hydrophilic cell surface select hydrophilic materials [14]. In our research, only the *P. mirabilis* C12 strain was found to be hydrophobic due to the affinity for *p*-xylene higher than 50%. The *P. mirabilis* ATCC 29906 and 1984 strains which were isolated from urine were hydrophilic, while the *P. mirabilis* C12 strain which was obtained from biofilm formed on a urinary catheter surface was hydrophobic. These results are in line with the thermodynamic theory.

As was suggested in the studies with *E. coli* [45], hydrophobicity could be characterized as a novel virulence mechanism of pathogens. Drumm et al. [50] noticed that the adhesion process is strongly related with the hydrophobic properties of *E. coli* cells. Similarly, CSH-mediated adherence to abiotic surfaces of another uropathogen, *Pseudomonas aeruginosa*, has been described in a few publications. According to Vanhaecke et al. [51] and Baumgarten et al. [52], the adherence of *P. aeruginosa* cells to stainless-steel and polystyrene surfaces is facilitated by surface hydrophobicity. In our investigation, the cells of the hydrophobic *P. mirabilis* C12 strain were defined as strongly adherent to the silicone surface, while those of the hydrophilic *P. mirabilis* 1984 and ATCC 29906 strains occurred to be moderately and weakly adherent, respectively. It is obvious that bacterial attachment to catheter surfaces is dependent upon the hydrophobicity of the microorganisms and the biocompatibility of the catheter surface. *P. mirabilis*-mediated colonization of non-treated silicone- or latex-based urological catheters, as well as those modified by hydrogel, silver nanoparticles embedded in hydrogel, 2 methacryloloxyethylphosphorylcholine co-polymerized with long-chain alkyl methacrylates, Zn-doped CuO nanoparticles, bacteriophages, eucalyptus essential oil, tea tree oil, terpinen, cineole or eugenol, have been repeatedly reported [53].

Instead of hydrophobicity, the zeta potential value may have importance for the adhesion of pathogens. The heterogeneous zeta potential of *Enterococcus faecalis* cells has been reported as support for bacterial adherence [54]. Czerwonka et al. [48] examined *P. mirabilis* strains whose strong adhesion was caused by more negative values of the zeta potential (PrK 61/57 strain), while weak adhesion was related to the less negative zeta potential (S1959 strain). On the other hand, there are reports which exclude the impact of surface electronegativity on the attachment of other uropathogens, including *Campylobacter jejuni* [55] and *E. coli* [56], to both biotic and abiotic surfaces. Besides the outer membrane charge, polarization of the inner membrane could play a significant role in the colonization of the catheter surface by bacteria. In our studies, the inner membranes of the *P. mirabilis* cells attached to the catheter surface were depolarized. According to the study by Manna et al. [57] and Benarroch et al. [58], cytoplasmic membrane depolarization could be caused by spatially propagating waves of the potassium ion (K^+^) during communication with distant members within the biofilm through long-range electrical signals. This theory has been confirmed by other authors. Prindle et al. showed that opening of the potassium channels, which triggers a relayed event of depolarization of neighboring cells, mediates electrical signaling within a *B. subtilis* biofilm [16]. Moreover, Lundberg et al. revealed that the deletion of the gene encoding a potassium ion channel prevents *B. subtilis* biofilm formation [17]. Although bacterial ion channels are suspected to have functional roles in signaling, the importance of the membrane potential for the urological catheter colonization by *P. mirabilis* bacteria remains unclear. Siciliano et al. [59] observed that membrane depolarization of rat hippocampal slices induced by an increase in the extracellular concentration of K^+^ ions resulted in tyrosine phosphorylation of two kinases involved in the adhesion process. However, these results cannot be related to bacterial cells.

A qualitative composition of *P. mirabilis* fatty acids shows a similarity to *E. coli*—the most common representative of *Enterobacteriaceae* [60]. In general, palmitic acid is the major fatty acid in the *P. mirabilis* membranes. Its content ranges from 47.3% to 61.0% of the total FAs amount, depending on the strain [18,19,20,21,61]. Our results showed a lower (~35%) C16:0 level in the *P. mirabilis* ATCC 29906, 1984 and C12 planktonic cells. A similar content of C16:0 (~29%) was observed by Dai et al. [62] in *P. mirabilis* KCTC 2566 membranes. The study by de Carvalho and de Fonseca [63] revealed that a rise in the summed content of fatty acids composed of more than 16 carbon atoms linearly increased microbial hydrophobicity. On the other hand, the research carried out by Moorman et al. [64] proved that cell surface hydrophobicity is intensified by a decrease in (1) the ratio of *anteiso/iso* fatty acids, (2) the ratio of C15/C17 and (3) the level of C18 unsaturated fatty acids (UFA). Margaric acid, similar to fatty acids in *anteiso* forms, was not identified in the membranes of the tested *P. mirabilis* strains. Other dependencies were not observed during our investigation. However, palmitoleic acid proved to be the component that reduced cell-surface hydrophobicity of the *P. mirabilis* ATCC 29906, 1984 and C12 strains. The studies with a different strain of *P. mirabilis* also revealed a lower C16:1 level in the membranes of the more hydrophobic strain R+ (containing R-plasmid RP1), compared to the R– strain [19,23]. Moreover, Lagha et al. [65] noted the same effect in the membranes of two *Salmonella enterica* strains with different hydrophobicity. It is still unclear why the elevated level of C16:1 weakens CSH.

It has also been reported that an increased amount of hydroxy fatty acids contributes to lower hydrophobicity of *Stenotrophomonas maltophilia* [66], while the presence of eicosapentaenoic acid in the membranes of both *Shewanella marinintestina* IK-1 [67] and *E. coli* DH5αEPA [68] results in greater surface hydrophobicity. Notwithstanding, the hydroxy fatty acids of the tested *P. mirabilis* strains were residual, and polyunsaturated fatty acids (PUFA) were not detected. Importantly, cell hydrophobic properties of the *P. mirabilis* ATCC 29906, 1984 and the C12 strains were promoted by the saturation degree of fatty acids. Similarly, the more hydrophobic *P. mirabilis* R+ (with R-plasmid RP1) strain was characterized by more saturated FAs than the R– strain [19,23], and the more hydrophobic S1 strain of *S. enterica* had a lower ratio of unsaturated to saturated fatty acids (UFA/SFA) than the S1 strain [65]. Furthermore, the degree of fatty acids saturation seems to be important not only for hydrophobic properties but also for the adhesion ability of microorganisms, especially probiotic bacteria whose basic feature is adhesiveness to intestinal epithelium. Indeed, in the more adhesive *L. rhamnosus* PEN strain, a higher saturation degree of FAs in comparison to the E/N strain was observed [12]. For other probiotic strains such as *Lactobacillus acidophilus, Lactobacillus casei* and *Bifidobacterium bifidum*, an increase in the UFA level resulted in the reduced adherence of the bacteria to human intestinal epithelial-like Caco-2 cells [11]. Moreover, it was noticed that cell PUFA content (especially docosahexaenoic acid) could modify the attachment of *Lactobacillus* [69]. Taking our results together, the role of fatty acids in supporting or inhibiting the *P. mirabilis* ATCC 29906, 1984 and C12 adhesion to a catheter surface could not be indicated.

It is known that elongation of the acyl chains, an increase in the saturation degree, isomerization *cis* to *trans* of unsaturated fatty acids and change of *anteiso* to *iso* of branched fatty acids affect the biophysical properties of the bacterial membrane, primarily reducing its fluidity and permeability [70]. However, the specific role of FAs in terms of bacterial hydrophobicity and adhesion ability is still poorly understood. In fact, fatty acid molecules are hydrophobic in nature. It is noteworthy that C16 and C18 acyl chains are able to reach approximately the middle of the membrane bilayer. Longer C24 chains should be able to reach farther beyond the bilayer [71], thus altering surface hydrophobicity. Moreover, extracellular free fatty acids immobilized on the cell are thought to be a determinant of surface properties [72]. Iimura et al. [73] reported that the removal of palmitoleic and oleic acids from the cell surface resulted in lowering microbial hydrophobicity to naught. Further experiments are required to discover the exact mechanism of this phenomenon. It is difficult to speculate at this point on the role of individual fatty acids (especially C16:1) in the reduction or promotion of the *P. mirabilis* cells’ hydrophobicity.

PE is a frequent and abundant phospholipid in Gram-negative bacteria. Our research showed that phosphatidylethanolamines constituted about 70% of the total phospholipids in the *P. mirabilis* planktonic cells. This is consistent with the previous papers focused on membrane composition of this pathogen [74,75]. Unfortunately, data on the composition of fatty acids incorporated into PE molecules are not available. The exception is palmitic acid being mentioned as the main component of PE [22] of the *Proteus* P18 strain (similar to *P. mirabilis*). The second class of PLs identified in *P. mirabilis* ATCC 29906, 1984 and C12 strains was PG, accounting for 30% of total phospholipids of planktonic cells. Other researchers reported that phosphatidylglycerols represented from 11% to 17% of total *P. mirabilis* lipids, depending on the strain and growth conditions [22,74]. As mentioned before, hydrophobicity of the studied *P. mirabilis* strain was reduced by a simultaneous change in the PG (up) and PE (down) levels. A decrease in the PG content has been described as a limitation for cell hydrophobicity of a nisin-resistant mutant of *Listeria monocytogenes* [76]. The phospholipid heads used to be perceived as structures contributing to the hydrophobicity of the *Vibrio cholerae* membrane [77]. However, the cause was not found. On the other hand, Hart and Champlin [78] questioned the significance of both PG and PE for the hydrophobic properties of *Pasteurella multocida* and *Actinobacillus lignieresii* cells.

In some studies, PE-dependent adhesion has been noticed, but the precise function of this principal bacterial phospholipid in the process remains unclear. Yu et al. [79] found that PE deficiency could impair *E. coli* adhesion on macrophages or glass coverslips. In other studies, a 95% decrease in the adhesion of a minus-PE *E. coli* mutant compared to the wild-type was noted [10]. Our results are in contradiction to these reports. The *P. mirabilis* ATCC 29906, 1984 and C12 cells attached to silicone surfaces were characterized by an at least 20% decrease in the amounts of synthesized PEs compared to the nonattached cells. Moreover, the adhesion of probiotic bacteria related to the PG content in their membranes was also mentioned [11]. On the other hand, Benamara et al. [80] described a drastic decrease in PLs containing a branched chain and/or a cyclopropyl chain in *P. aeruginosa* cells during the adherence to glass wool.

The nature of PLs head-group, as well as the length and saturation of its acyl chains, affect the structural features of the membrane bilayer, such as curvature, electrostatic charge and viscosity [81]. Contrary to the outer membrane lipids, the inner membrane lipids could have no direct effect on the interactions between the bacterial cells and various surfaces. However, membrane lipid composition is considered an important determinant of the cell size and shape as well as an essential morphological parameter that ensures surface adhesion and biofilm formation [10]. Moreover, it was assumed that a major proportion of straight FAs is functional for the interaction of membrane phospholipids, or their specific loci, with the cell-surface proteins that mediate the contact of the cell envelopes with the abiotic surfaces [72]. On the other hand, the role of individual lipids of the studied *P. mirabilis* strains in supporting CSH (PE 32:0, PE 33:0, PE 38:2, PG 33:1, PG 34:0), reducing CSH (PE 30:1, PE 32:1, PG 30:2) or promoting adhesion (PE 37:2) is still difficult to define.

Biofilm-associated cells exhibit reduced growth rates, distinct physiological characteristics and altered gene expression compared to their planktonic counterparts, probably because the encapsulated bacteria in the biofilm have a decreased nutrient and oxygen supply leading to a decreased metabolic rate [82]. However, such a description concerns a biofilm lasting at least a few days, and therefore, it can be supposed that the impact of the 24 h biofilm architecture on the activity of cellular metabolism of *P. mirabilis*, including the lipid synthesis pathways, is unnoticeable. The ability of cells grown in a biofilm to alter membrane lipid composition is one of the crucial characteristics for *C. albicans* biofilm development. Biofilm-associated yeast cells display phase-dependent changes in phospholipid classes and in the levels of lipid raft formation [83]. Pisithkul et al. [84] performed a time-resolved analysis of the metabolic changes associated with *B. subtilis* biofilm formation and development. The authors proved that the transition from the biosynthesis of fatty acids and phospholipids to fatty acid β-oxidation occurred between the 12th and 16th hour of biofilm development. Moreover, during early biofilm development, the fatty acid synthesis pathway displayed distinct downregulation with concurrently strong upregulation of fatty acid β-oxidation. The alterations in fatty acid metabolism indicate a possible reconstruction of the lipid membrane composition during *B. subtilis* biofilm development [84].

Laurdan has a large excited state dipole moment, hence its ability to report the extent of water penetration into the membrane bilayer, which is in turn associated with density of lipid packing and membrane fluidity. The emission spectrum of this membrane dye within a single phospholipid bilayer is centered at 490 nm when the lipids are in a disordered phase and is shifted to the blue (around 440 nm) when the lipids are in a more packed phase. A calculation of the GP value enables a quantitative way to measure the emission shift [85]. GP values in liquid phase membranes typically range from −0.3 to 0.3, and those in the gel phase range from 0.5 to 0.6. Our results achieved during the *P. mirabilis* cells’ examination were within the above range. We found that the bacterial cells attached to the catheter surface had less packed, liquid phase of their membranes in comparison to tightly packed membranes of the planktonic cells.

Membrane fluidity has been classically considered a key physical property affecting cell adhesion. Matsuzaki et al. [86] described reduced adherence of human stem cells in the presence of polyphenols, which was associated with higher GP values and a more ordered membrane of all free germ layers. The authors pointed to the impeded transport of adhesive proteins onto the cell surface because of stiffened membranes as the cause of the observed phenomenon [86]. We obtained similar results. The studied *P. mirabilis* cells recovered from the colonized catheter had more liquid membranes than the planktonic cells. It draws attention to the significance of optimum membrane fluidity for easy lateral membrane protein diffusion, which reflects the adhesion characteristics. On the other hand, it seems possible that the *P. mirabilis* cells located in the immediate vicinity of the catheter trigger cellular mechanisms, leading to the rearrangement of the membrane composition in order to facilitate the secretion of adhesion proteins to the cell surface.

The generalized polarization values can be used as a reporter for head group density and fatty acyl spreading. We found that membrane fluidization of adherent *P. mirabilis* ATCC 29906, 1984 and C12 cells was attributable to high levels of both unsaturated fatty acids and phosphatidylglycerols. However, it cannot be excluded that modifications in other cell components, such as proteins, also affected the GP value during the initial adhesion.

At present, fatty acids’ interaction with phospholipids is thought to be a facilitation of the contact between the cell envelopes and abiotic surfaces [71]. However, the analyses at a molecular level are required to verify this hypothesis. Surprisingly, the vaccines and small molecules targeting bacterial adhesion [4] to weaken colonization and prevent biofilm formation have already been applied. It is seen that this approach would be helpful for catheterized patients.

In the future, we plan to expand the research to a larger number of clinical *P. mirabilis* strains to relate the obtained results with species and not strain features. Further research will include one more virulence factor responsible for ascending infections, i.e., swarming motility of *P. mirabilis* cells on a silicone catheter surface. Results of the designed studies will help to resolve the question of whether the observed changes between adherent cells and planktonic cells’ properties may be attributed to the modifications of the membrane composition of swarmer and planktonic cells. Due to the fact that a particularly striking feature of *P. mirabilis* is its swarming motility, in which elongated swarmer cells form multicellular rafts and move rapidly over solid surfaces, swarming on catheter surfaces could be biologically relevant and cannot be omitted. Molecular studies will focus on cardiolipins, sphingolipids, quantitative analysis of secreted extracellular polymeric substances and qualitative analysis of produced biosurfactants.

## 5. Conclusions

The significance of fimbriae, non-fimbriae adhesins and LPS in the mediation of the microbial adhesion process is well-recognized. In this study, cell-surface hydrophobicity, the inner membrane potential and cell membrane fluidity were also proven to play important roles. Our research showed that the bacterial adherence is supported by strong hydrophobic properties of the cell surfaces and that the mechanism of the cell adhesion to a silicone catheter surface requires the depolarization of *P. mirabilis* inner membranes and reduction of stiffness and packing density of membrane components, which could facilitate diffusion of adhesins onto the cell surface. Moreover, this paper provided evidence that differentiation in both GP and CSH values is attributable to differences in PLs and FAs composition.

## Data Availability

Not applicable.

## Supplementary Materials

The following are available online at https://www.mdpi.com/article/10.3390/ijms22168452/s1.
