# Supplementary material for: Phospholipids and Fatty Acids Affect the Colonization of Urological Catheters by Proteus mirabilis
Source: Int J Mol Sci. 2021 Aug 6;22(16):8452. doi: 10.3390/ijms22168452 (PMC8395112; doi:10.3390/ijms22168452)
Supplement: Supplementary file 1 [file ijms-22-08452-s001.zip › ijms-1279925-supplementary.pdf]

SUPPLEMENTARY MATERIAL

Phospholipids and fatty acids affect the colonization of urological catheters by *Proteus mirabilis*

Paulina Stolarek[1]*, Przemysław Bernat[2], Dominika Szczerbiec[1], Antoni Różalski[1]

[1] Department of Biology of Bacteria, Faculty of Biology and Environmental Protection, University of Lodz, Banacha 12/16, 90-237 Lodz, Poland

[2] Department of Industrial Microbiology and Biotechnology, Faculty of Biology and Environmental Protection, University of Lodz, Banacha 12/16, 90-237 Lodz, Poland

E-mail addresses: paulina.stolarek@biol.uni.lodz.pl; przemyslaw.bernat@biol.uni.lodz.pl; dominika.szczerbiec@biol.uni.lodz.pl; antoni.rozalski@biol.uni.lodz.pl

* Corresponding author: Tel: +48 42 635 43 24, Fax: +48 42 665 58 18, e-mail: paulina.stolarek@biol.uni.lodz.pl

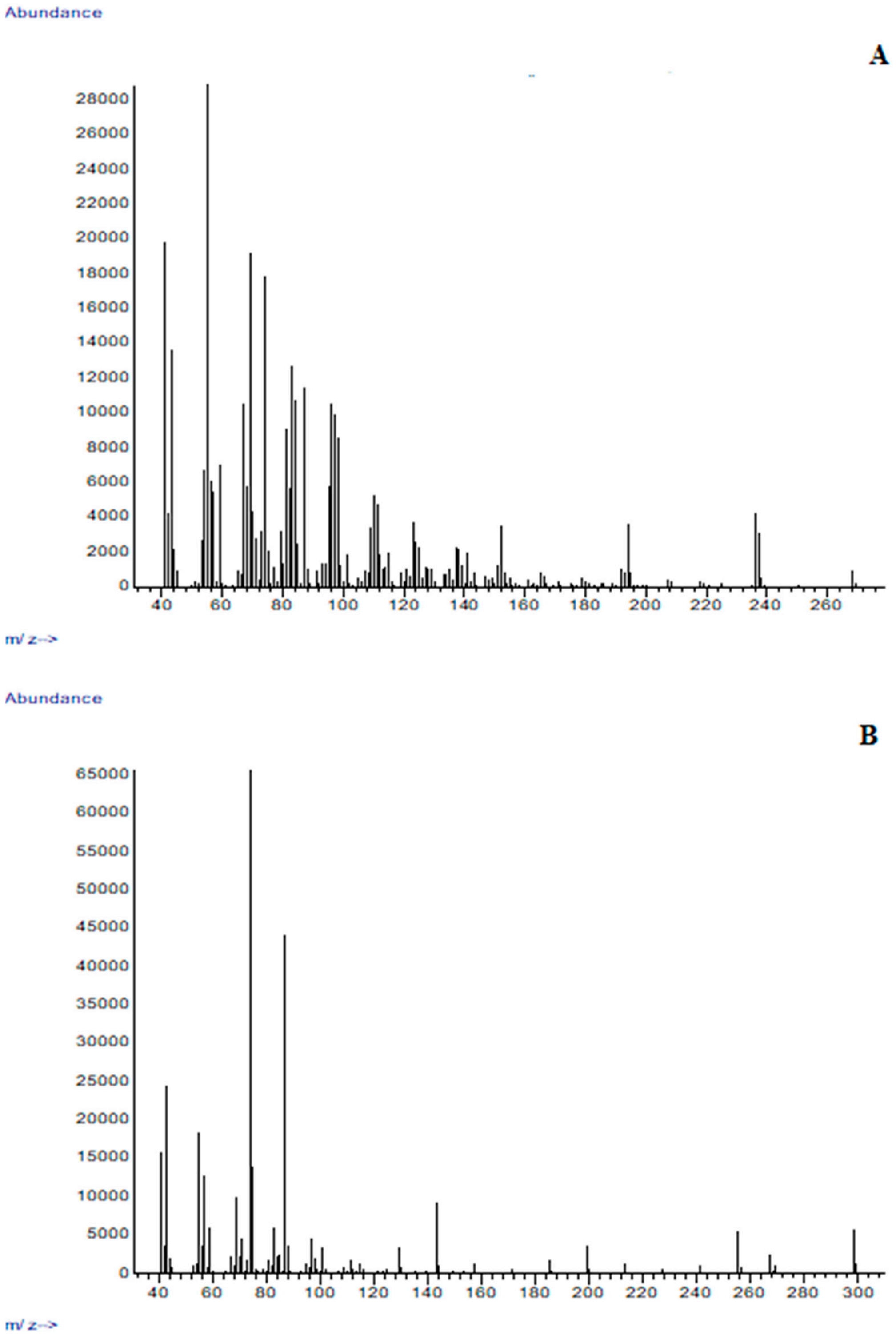

Figure S1. GC-MS mass spectra of palmitoleic acid (A) and stearic acid (B).

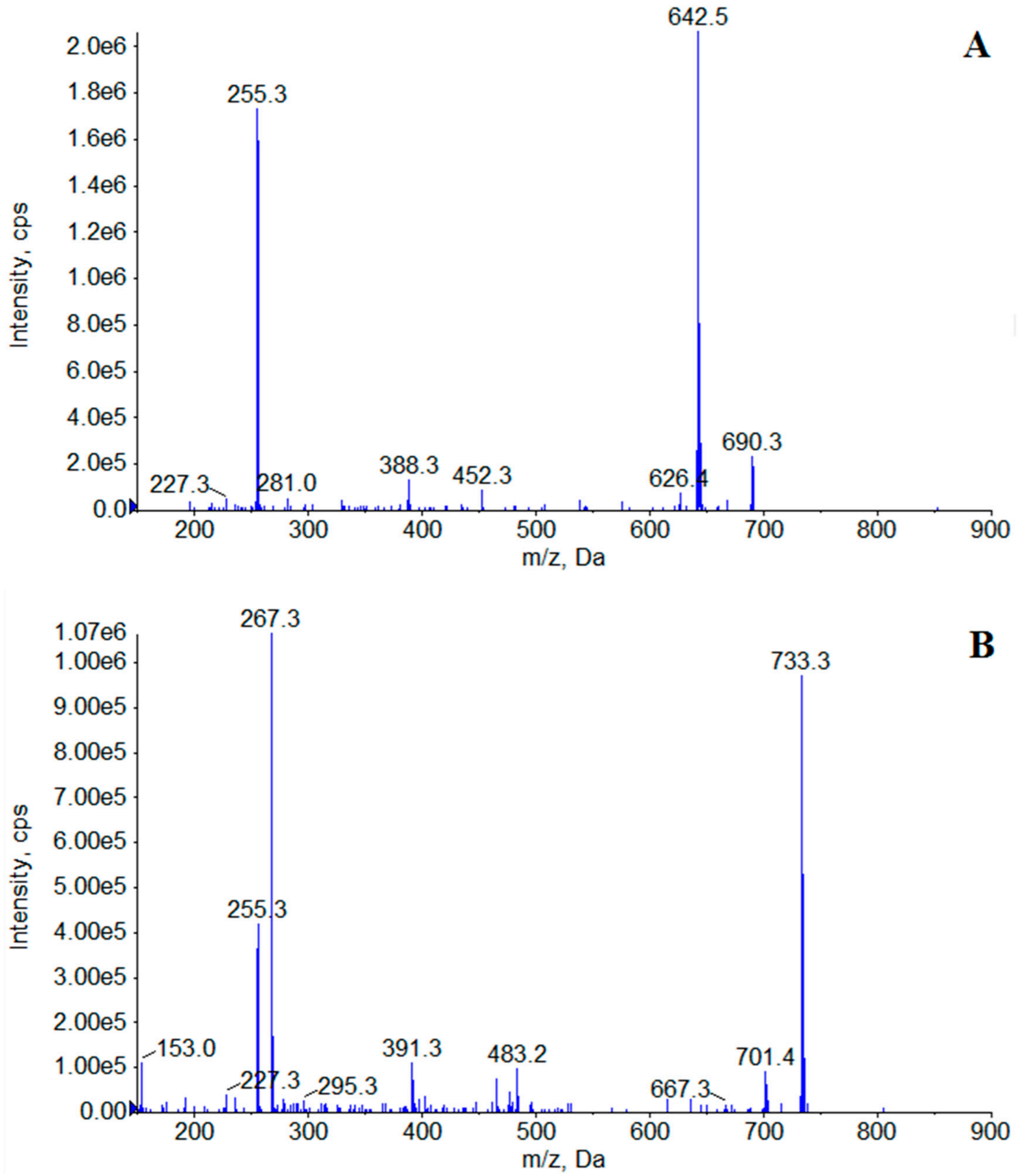

Figure S2. LC-MS/MS mass spectra of PE 32:0 (A) and PG 33:1 (B).

Table S1. A Spearman's correlation between fatty acids or phospholipids of the *P. mirabilis* strains and their cell-surface hydrophobicity or ability of adhesion.

	Variable 1.	Variable 2.			
		adhesion		hydrophobicity	
		correlation coefficient [r]			
		6-hour cells	24-hour cells	6-hour cells	24-hour cells
**Fatty acids**	C11:0	-0.85	-0.91	–	–
	cis C16:1	–	–	-0.99	-0.73
	FAs saturation degree	–	–	0.58	0.99
**Phospholipids**	PE 30:1	–	–	-0.82	-0.68
	PE 32:0	–	–	0.95	0.55
	PE 32:1	–	–	-0.98	-0.90
	PE 33:0	–	–	0.96	0.96
	PE 37:2	0.63	0.99	–	–
	PE 38:2	–	–	0.98	0.95
	PG 30:2	–	–	-0.99	-0.99
	PG 33:1	–	–	0.99	0.99
	PG 34:0	–	–	0.73	0.83
	Σ unsaturated PG's	-0.50	-0.99	-0.94	-0.91
	PG/PE	-0.74	-0.75	-0.94	-0.89
	DBI	–	–	-0.99	-0.88

Only correlation with r ≥ 0.50 is presented.
